# Histone deacetylase inhibitor vorinostat suppresses the growth of uterine sarcomas *in vitro *and *in vivo*

**DOI:** 10.1186/1476-4598-9-49

**Published:** 2010-03-04

**Authors:** Andelko Hrzenjak, Farid Moinfar, Marie-Luise Kremser, Bettina Strohmeier, Edgar Petru, Kurt Zatloukal, Helmut Denk

**Affiliations:** 1 Lore Saldow Research Unit for Molecular Pathology of Gynecologic Tumors, Department of Pathology, Medical University of Graz, Auenbruggerplatz 25, 8036 Graz, Austria; 2 Institute of Internal Medicine, Department of Pulmonology, Lung Cell Laboratory, Medical University of Graz, Auenbruggerplatz 20, 8036 Graz, Austria; 3 Department of Obstetrics and Gynecology, Medical University of Graz, Auenbruggerplatz 14, 8036 Graz, Austria

## Abstract

**Background:**

Uterine sarcomas are very rare malignancies with no approved chemotherapy protocols. Histone deacetylase (HDAC) inhibitors belong to the most promising groups of compounds for molecular targeting therapy. Here, we described the antitumor effects of suberoylanilide hydroxamic acid (SAHA; vorinostat) on MES-SA uterine sarcoma cells *in vitro *and *in vivo*. We investigated effects of vorinostat on growth and colony forming ability by using uterine sarcoma MES-SA cells. We analyzed the influence of vorinostat on expression of different HDACs, p21^WAF1 ^and activation of apoptosis. Finally, we examined the antitumor effects of vorinostat on uterine sarcoma *in vivo*.

**Results:**

Vorinostat efficiently suppressed MES-SA cell growth at a low dosage (3 μM) already after 24 hours treatment. Decrease of cell survival was even more pronounced after prolonged treatment and reached 9% and 2% after 48 and 72 hours of treatment, respectively. Colony forming capability of MES-SA cells treated with 3 μM vorinostat for 24 and 48 hours was significantly diminished and blocked after 72 hours. HDACs class I (HDAC2 and 3) as well as class II (HDAC7) were preferentially affected by this treatment. Vorinostat significantly increased p21^WAF1 ^expression and apoptosis. Nude mice injected with 5 × 10^6 ^MES-SA cells were treated for 21 days with vorinostat (50 mg/kg/day) and, in comparison to placebo group, a tumor growth reduction of more than 50% was observed. Results obtained by light- and electron-microscopy suggested pronounced activation of apoptosis in tumors isolated from vorinostat-treated mice.

**Conclusions:**

Our data strongly indicate the high therapeutic potential of vorinostat in uterine sarcomas.

## Background

Uterine sarcomas are uncommon, representing approx. 5% of all uterine malignancies [1]. These tumors are often diagnosed in advanced stages and carry an unfavorable prognosis. The final diagnosis is based upon histological and immunohistochemical analyses of tumor tissue obtained by biopsy or surgical excision [2]. Due to the low incidence of uterine sarcomas, data concerning both molecular mechanisms of their pathogenesis and therapeutic approaches are quite limited and further information is needed. Since uterine sarcomas are rare, they are also not uniformly treated. The mechanisms involved in the tumorigenesis are only in the beginning of being elucidated. Thus, the establishment of *in vivo *systems for basic investigations and testing therapeutic approaches in uterine sarcomas is particularly important. Cell lines originating from these malignancies are rare and so are *in vivo *systems. The usefulness of some uterine sarcoma cell lines is limited by the fact that the vast majority of them are not tumorigenic in nude mice. This is also the case for cell lines isolated from low grade endometrial stromal sarcomas, e.g., ESS-1 cells [3]. For some other cell lines details regarding tumorigenicity in nude mice are missing. In a recent publication Kakuno *et al *reported the establishment of a new cell line (OMC-9) originated from a human endometrial stromal sarcoma [4]. According to the authors, these cells are tumorigenic in nude mice and could, therefore, be useful for development of an *in vivo *system. Unfortunately, this cell line was not commercially available till now. Since MES-SA cells established by Harker and coauthors are tumorigenic in nude mice, we decided to use them both for *in vitro *and for *in vivo *experiments in order to test the efficacy of suberoylanilide hydroxamic acid (SAHA; vorinostat).

Vorinostat is a potent inhibitor of HDACs class I and II. These enzymes are responsible for deacetylation of histones and some other proteins and consequently control the expression of different regulatory genes which are responsible for cell growth, proliferation, apoptosis, autophagy and for regulation of other mechanisms involved in the tumor development and growth [5-11]. Our recent data, both published and unpublished, strongly suggest that some HDACs are deregulated in endometrial stromal sarcomas and other uterine tumors of mesenchymal origin [12,13]. The therapeutic utility of vorinostat is supported by the fact that it has been recently approved by FDA for therapy of cutaneous T-cell lymphoma. Moreover, vorinostat is used in clinical trials in patients with other solid tumors, such as mesothelioma, medulloblastoma, prostate and thyroid cancer [14-16]. Our *in vitro *and *in vivo *data suggest that vorinostat is an active drug potentially suitable for targeted treatment of uterine sarcomas.

## Methods

### Chemicals and cell lines

All chemicals and media were purchased from Sigma (SIGMA-ALDRICH Handels GmbH, Vienna, Austria), unless otherwise specified. Vorinostat was purchased from Alexis Biochemicals (Lausen, Switzerland). The human uterine sarcoma cell line MES-SA, established by Harker *et al *[1], was purchased from ATCC (ATCC Nr. CRL-1976). The original specimen was characterized as poorly differentiated uterine sarcoma and the cells were isolated after hysterectomy of a 56 years old Caucasian woman. It has been also shown that these cells are highly tumorigenic in nude mice. All experiments were performed according to local ethical guidelines.

### Drug formulation

For in vitro experiments a 10 mM vorinostat stock solution was prepared with DMSO and stored at -20°C. Since it is well known that DMSO can cause different inflammatory reactions when injected intraperitoneally for a longer period of time, we wanted to avoid this solvent for our *in vivo *experiments. Therefore, we prepared a solution of vorinostat in HOP-β-CD (2-hydroxypropyl-β-cyclodextrin) as already described by Hockly *et al *[17]. Briefly, vorinostat was dissolved in 5 molar equivalents of HOP-β-CD in water, it was heated until fully dissolved, rapidly cooled on ice to room temperature and stored at -20°C. A fresh solution was prepared every week and administered to the mice by intraperitoneal injection in a total volume of approx. 300 μl, so that the final concentration for each animal was 50 mg/kg/day.

### *In vivo *experiments

The animal experiments were approved by the Austrian ministry of education and science according to the regulations for animal experimentation. Athymic Nude-Foxn1^nu/nu ^mice used in the present study were purchased from Harlan (Harlan Italy, San Pietro al Natisone, Italy). They were housed at 22°C at a constant light-dark cycle (12-h light, 12-h dark) and had free access to water and rodent chow (4-5% fat, 21% protein; Sniff, Soest, Germany). All animals used in this study were kept under standardized, pathogen-free living conditions in the animal facility of our department.

Twelve weeks old male mice (n = 14) were anesthetized with Isofluran (Pharmacia & Upjohn SA, Guyancourt, France) and 5 × 10^6 ^MES-SA cells were injected subcutaneously into the right flank of the animal. Mice from a control group received placebo containing 300 μl of empty HOP-β-CD (2-hydroxypropyl-β-cyclodextrin) vesicles. Another group of mice received vorinostat dissolved in HOP-β-CD at a concentration of 50 mg/kg/day. Both, empty vesicles and vorinostat were administered intraperitoneally, starting on the day 4 after the injection of MES-SA tumor cells. Mice body weight and tumor size (w^2 ^× l × 0.52; measured by caliper) were estimated twice a week. All mice were treated for 21 days and afterwards sacrificed by cervical dislocation. Each tumor was isolated as a whole and different tumor parameters (weight, volume, size and macroscopic appearance) were determined. Finally, tumor slices were cryo preserved and formalin fixed (4%) for further analyses.

### Western blot analysis

Cell lysates were prepared by using RIPA buffer (25 mM Tris-HCl pH 7.6, 150 mM NaCl, 1% NP-40, 1% sodium deoxycholate, 0.1% SDS), and the protein concentration was determined by Bio-Rad D_C _Protein Assay (Bio-Rad Laboratories, Hercules, CA). Protein lysates were separated by SDS-PAGE and transferred to nitrocellulose membrane (Bio-Rad). Following antibodies and dilutions were used: rabbit anti HDAC1 (1 μg/ml) (Labvision, Fremont, CA, USA); rabbit anti HDAC2 (1 μg/ml) (Zymed, San Francisco, CA, USA); rabbit anti HDAC3 (9 μg/ml) (Novus biologicals, Littleton, CO, USA); rabbit anti HDAC7 (3 μg/ml) (Abcam, Cambridge, UK); mouse anti p21^WAF1 ^(0.5 μg/ml) (Zymed). As secondary antibodies we used rabbit anti-mouse and swine anti-rabbit HRP-coupled antibodies at a final concentration of 1 μg/ml (DAKO, Copenhagen, Denmark). An overnight incubation at 4°C was used for all primary antibodies, followed by washing and 2-hours incubation at RT with secondary antibodies. Specific protein bands were visualized by enhanced chemiluminescence assay (ECL; Amersham Biosciences, Buckinghamshire, England). To demonstrate equal loading of protein samples all western blots were probed for β-tubulin.

### Clonogenic assay

MES-SA cells were seeded in ∅ 6 cm culture dishes (300 cells/dish) and treated with 3 μM vorinostat for 24, 48 and 72 hours. Afterwards fresh medium was added and the cells were cultured for another 14 days followed by fixation with butanol:acetic acid (3:1) and staining with 0.5% crystal violet.

### Electron microscopy

Cryo-preserved tumor tissue was fixed with ice-cold glutaraldehyde (2.5% in 0.1 M cacodylate buffer, pH 7.4) for 30 minutes. After fixation, the samples were postfixed in 1% OsO_4 _in the same buffer for 30 min, washed twice with cacodylate A buffer and rehydrated through series of increasing alcohol concentrations (70, 80, 90, 95% ethanol, 10 min each). Tissue was incubated in prophylenoxid - epoxid resin (1:1) for 1 hour and afterwards with epoxid resin over night at 4°C. Ultrathin sections were stained with uranyl acetate and lead citrate and viewed with a Philips CM100 transmission electron microscope. Photographs were made with Kodak Electron Image Film SO-163 (Kodak, Vienna, Austria) and developed following the procedure recommended by producer.

### Apoptosis measurement

MES-SA cells were treated with medium containing 3 μM vorinostat for indicated time periods. After harvesting the cells were fixed in 2% formaldehyde for 10 min at 37°C, followed by permeabilization with methanol (90%). Staining was performed by Cleaved Caspase-3 (Asp175) antibody conjugated with Alexa Fluor 488 (Cell Signaling Technology, #9669) for 60 minutes at room temperature. Measurements were performed on FACSCalibur™ (BD Biosciences). Staurosporine-treated cells (1 μM for 4 hours) were used as positive and untreated MES-SA cells as negative controls.

### Statistical analysis

If not stated otherwise, all values represent means of at least three independent experiments ± SD. Values were compared using Student's *t *test. *P *≤ 0.05 was considered statistically significant.

## Results

### Vorinostat inhibits cell growth and colony formation *in vitro*

**Figure 1:**
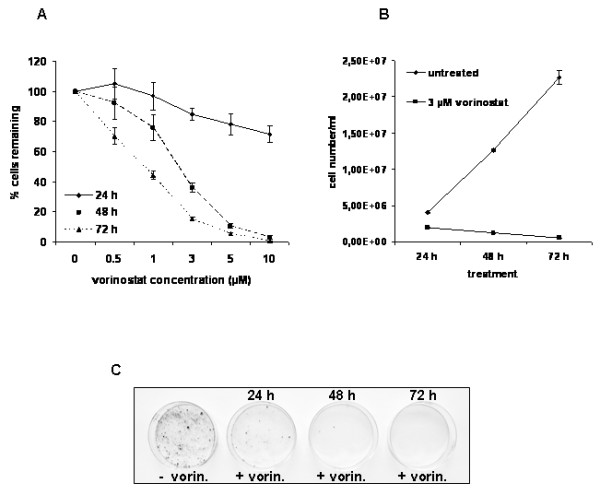
**Vorinostat decreases the growth and colony forming ability of MES-SA cells *in vitro***. (a) Dose response curve for MES-SA cells treated with five different vorinostat concentrations over three time periods. Cell proliferation was measured by using [^3^H]thymidine uptake assay. Results are expressed as percentage inhibition of [^3^H]thymidine incorporation upon vorinostat treatment and normalized to untreated cells. (b) MES-SA cells were treated with 3 μM vorinostat for 24, 48 and 72 hours. Vorinostat induced a strong decrement in the number of treated cells in comparison to untreated control cells. (c) The ability of colony formation was drastically reduced in vorinostat-treated MES-SA cells already after 24 hours and continued to decrease in a time-dependent manner.

In order to examine the most effective vorinostat concentration for growth inhibition of MES-SA uterine sarcoma cells, we performed cell proliferation experiments by using [^3^H]thymidine uptake assay. Five different vorinostat concentrations (0.5, 1, 3, 5, and 10 μM) were tested over three time intervals (24, 48, and 72 h). As shown in Fig. 1A, vorinostat concentration as low as 0.5 μM caused growth inhibition in comparison to untreated, control MES-SA cells. These effects largely increased with higher vorinostat concentrations. Considering a dose response curve, 3 μM vorinostat has been used as a working concentration for further experiments, this concentration being in agreement with our previous studies on ESS-1 cells [13]. As shown in Fig. 1B, the growing curve for MES-SA cells treated with 3 μM vorinostat showed a prominent decrease in comparison to untreated cells. Twenty four hours after the vorinostat treatment cell number was decreased to 48% in comparison to untreated cells, whereas 48 and 72 hours after treatment it was further decreased to 9% and 2%, respectively. These data clearly indicate high sensitivity of MES-SA cells to vorinostat.

For colony forming assay, 300 MES-SA cells were seeded per ∅ 6 cm dish. After the treatment with vorinostat for 24, 48 and 72 hours they were grown for another 14 days and finally stained with crystal violet. As can be seen in Fig. 1C, there was a pronounced difference in the colony formation ability between untreated and treated MES-SA cells. This reduced number of colonies showed that vorinostat efficiently killed the cells in a time dependent-manner.

For more general impact of our findings, we additionally compared the MES-SA with ESS-1 endometrial stromal sarcoma cells. This was considered to be of further importance because no *in vivo *system for investigation of endometrial stromal sarcoma has been established until now. As can be seen in Table 1, these two cell lines share many similarities regarding the expression of cell-markers tested in our study. In addition, our previous data showed that vorinostat efficiently inhibits the growth of ESS-1 cells *in vitro *(13).

**Table 1:** Immunohistochemical characterization of MES-SA cells and comparison to the endometrial stromal sarcoma cell line ESS-1

Marker	Cell line	
	MES-SA	ESS-1
CD-10	-	+
HDAC-2	+++	+++
Caldesmon	-	-
Vimentin	+++	+++
Keratin	-	+ (5-10%; cytoplasmic)
EGFR	-	+++

-, negative; +, weakly positive; ++, moderately positive; +++, strongly positive immunohistochemical reaction.

### Vorinostat deregulates expression of HDACs and p21^WAF1^

**Figure 2:**
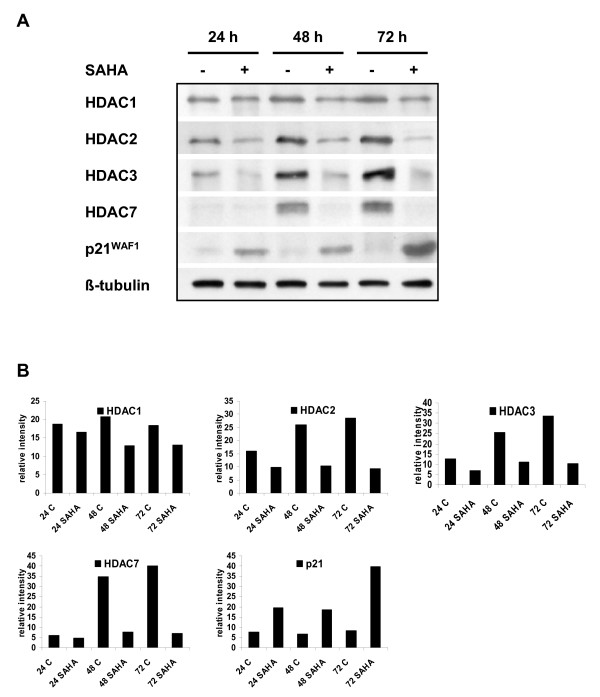
**Expression of different histone deacetylases and p21^WAF1 ^in untreated and vorinostat-treated MES-SA cells**. (a) Immunoblot analyses were used to examine the expression of different HDACs from class I (HDAC 1, 2 and 3) and class II (HDAC7). Note that not all HDACs were equally affected by vorinostat. HDAC2, 3 and 7 were highly deregulated, whereas HDAC1 was not affected. The expression of cyclin-dependent kinase inhibitor p21^WAF1 ^was highly increased in vorinostat-treated MES-SA cells. (b) Western blot data were quantified densitometrically and beta-tubulin was used as a loading control.

We examined the expression of different members of HDACs class I (HDAC1, 2 and 3) and class II (HDAC7) in untreated and vorinostat treated MES-SA cells. There was no difference in the HDAC1 expression in untreated and treated cells during the whole duration of treatment (72 hours in total) (Fig. 2A, B). On the other hand, HDAC2, 3 and 7 showed pronounced inhibition of expression by vorinostat. The expression of HDAC2 and HDAC3 was affected already 24 hours after the vorinostat treatment, whereas inhibition of HDAC7 was not detectable until 48 hours after starting the treatment. All effects concerning HDAC 2, 3, and 7 became even more pronounced after 72 hours of treatment.

The expression of a cyclin-dependent kinase inhibitor p21^WAF1 ^in vorinostat treated MES-SA cells was significantly upregulated already 24 hours after starting the treatment and continuously increased during the following 48 hours. These data are in agreement with previous results obtained with ESS-1 cells, suggesting a typical G1 arrest caused by the vorinostat treatment [12].

### Vorinostat inhibits tumor growth in xenograft mice

**Figure 3:**
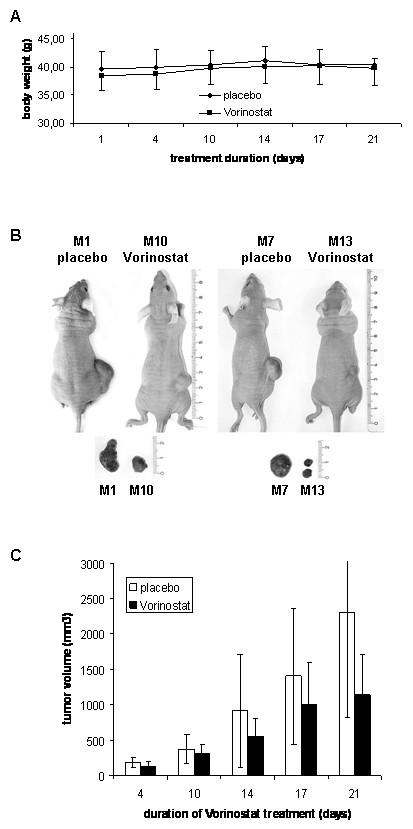
**Vorinostat suppressed tumor growth in nude mice xenografts**. 5 × 10^6 ^MES-SA cells were injected subcutaneously in Nude-Foxn1^nu/nu ^mice (n = 14) and four days later mice were injected either placebo (HOP-β-CD) or 50 mg/kg/day vorinostat dissolved in HOP-β-CD for a total of 21 days. (a) The mice weight curve was stable during the whole treatment indicating that no cachexy was induced by tumor growth. (b) Two representative xenograft mice from each placebo and vorinostat-treated group are shown. Tumors were isolated and tumor weight and volume were determined. In most cases tumors were monolobular, whereas only in two mice two tumor nodules were found at injection side. Obvious differences in tumor volumes were observed between the placebo (2304.7 mm^3^) versus the vorinostat-treated group (1135.4 mm^3^). (c) These differences were statistically highly significant at the end of treatment (*P *= 0.044).

On the fourth day after the subcutaneous injection of 5 × 10^6 ^MES-SA cells in all mice a small tumor was palpable. Thus, we decided to start with the vorinostat treatment at that time point. Mice were injected with either placebo (HOP-β-CD) or 50 mg/kg/day vorinostat for 5 consecutive days during the week, for 21 days. During the vorinostat treatment the body weight of all animals was determined twice a week and as shown in Fig. 3A, remained stable during the whole treatment period. This suggested that at indicated concentration and administration regimen vorinostat was well tolerated.

After 21 days of treatment the animals were sacrificed and different organs, i.e. liver, spleen, lung, heart and intestine, were checked for pathological changes and tumor metastases. No structural changes were found in analyzed organs. Tumors were isolated and tumor size, volume and weight were determined. Most tumors were encapsulated and well circumscribed. On day 21, there was a tumorous ulceration in one placebo case. Although the surface area of the tumor was well supplied with blood vessels, we observed tumors larger then 800 mm^3 ^to be most often necrotic in their center. However, this can be expected from the rapid tumor growth and inadequate blood supply of deeper tumor portions. In Fig. 3B the representative samples of two different tumors from each group (placebo and vorinostat-treated, respectively) are shown. Although the standard deviation of tumor size within groups, both placebo and treated one, was noticeable all over the treatment duration, at the end of treatment there was a significant difference (*P *= 0.044) in tumor volume between placebo and vorinostat-treated group. The average tumor volume in untreated mice was 2304.7 mm^3^, whereas in vorinostat-treated mice it was 1135.4 mm^3^. This difference represented a more than 50% reduction in comparison to the placebo group (Fig. 3C).

### Vorinostat induces apoptosis in MES-SA cells *in vitro *and *in vivo*

**Figure 4:**
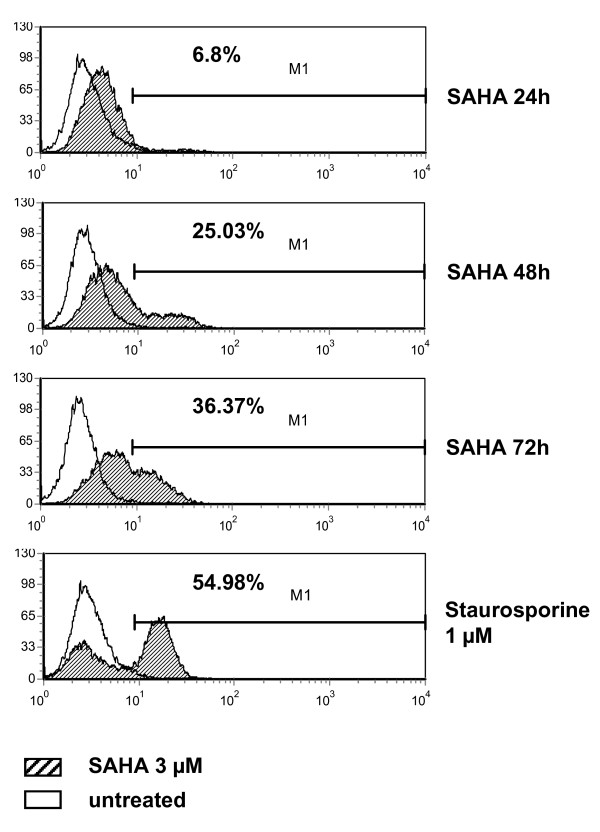
**Vorinostat induces apoptosis in MES-SA cells**. FACS analysis of MES-SA cells labelled with cleaved caspase-3 antibody conjugated with Alexa Fluor^® ^488 showed an increased percentage of apoptotic cells in vorinostat-treated cells, correlating with treatment duration. MES-SA cells treated with 1 μM staurosporine for 4 hours were used as a positive control. For numerical analysis two independent experiments, each containing duplicates, were performed. Representative data are shown.

We also performed flow cytometric analysis of MES-SA cells *in vitro*, untreated or vorinostat-treated, using Cleaved Caspase-3 (Asp175) Antibody (Alexa Fluor^® ^488 Conjugate). Vorinostat-treated cells clearly showed increased accumulation of apoptotic cells in a time dependent manner. After 72 hours of vorinostat treatment, the fraction of apoptotic cells reached 36.37% (Fig. 4).

**Figure 5:**
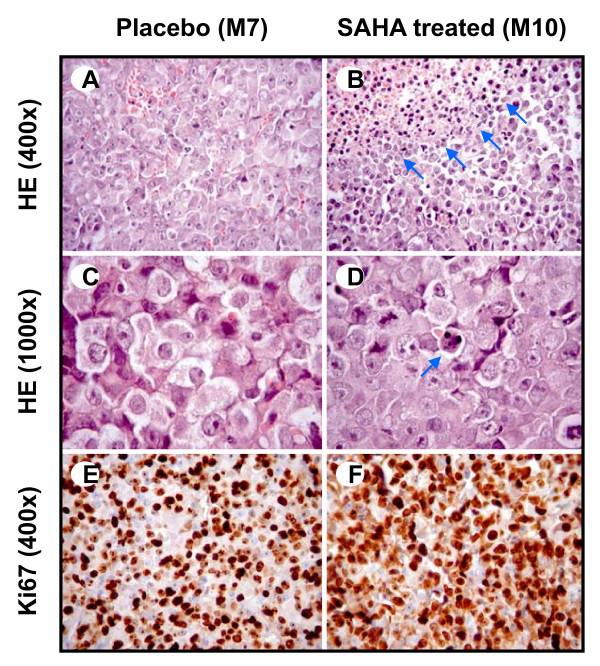
**H&E and Ki-67 immunostaining of tumor tissue from nude mice xenografts**. (a, c) Solid aggregates of highly atypical tumor cells showing large, round nuclei, prominent nucleoli and abundant cytoplasm were visible in tumors isolated from the placebo group. (b) In vorinostat-treated tumors, highly atypical tumor cells associated with abundant apoptosis were seen. Areas of apoptosis (marked by arrows) show tumor cells with smaller, dark nuclei with condensed chromatin and small rim of cytoplasm. Note the presence of vacuolated cytoplasm, fragmented tumor cells and isolated nuclei of tumor cells. (d) Aggregate of tumor cells with an apoptotic body characterized by dense eosinophilic cytoplasm and hyperchromatic nuclear fragments (marked by arrow). (e, f) More than 95% of tumor cells showed a positive nuclear immunoreaction for Ki-67, both in placebo and in vorinostat-treated tumors.

**Figure 6:**
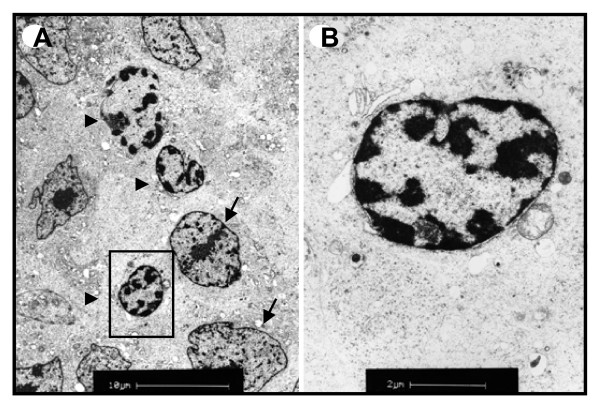
**Representative transmission electron microscopy of tumor tissue isolated from vorinostat-treated mouse xenografts**. (a) Shrunken nuclei with condensed and fragmented chromatin (arrow heads) in comparison to non-apoptotic nuclei (arrows). (b) Micrograph of the boxed area of panel A at higher magnification.

Hematoxylin-eosin (HE) staining showed the presence of prominent apoptotic figures in vorinostat-treated tumors, in contrast to tumors isolated from placebo group containing less apoptotic cells (Fig. 5A-D). Immunostaining for Ki-67 proliferation marker showed no significant differences between tumors isolated from placebo and vorinostat-treated group. Both groups were characterized by intense Ki-67 nuclear staining in 95% of tumor cells, indicating a very high proliferation rate (Fig. 5E, F), which is in accordance with the fast tumor growth observed. Condensed chromatin was also detected by electron microscopy in a large number of cells in tumors isolated from vorinostat-treated mice (Fig. 6A, B). These findings additionally indicate increased apoptotic activity upon vorinostat treatment.

## Discussion

Uterine sarcomas are very rare malignancies with poor prognosis. Precise diagnosis is usually made late, these tumors frequently grow highly aggressive and are resistant to chemotherapy. Thus, surgical excision is often the only treatment option [18,19]. Molecularly targeted therapies of different tumor types showed a promising improvement in the last few years. Histone deacetylases, a group of enzymes responsible for epigenetic changes of histones and some other proteins, belong to the most promising targets. Some inhibitors of these enzymes are already used in preclinical and clinical trials. Vorinostat efficiently inhibits HDACs class I and II by binding to the active site of the enzyme [20]. However, vorinostat seems to have different effects depending on the cell line used. While in most experimental systems vorinostat caused apoptotic changes, there are also data showing that autophagic processes are activated by vorinostat [13,21-23]. Vorinostat has been already approved by FDA for the therapy for cutaneous T-cell lymphoma [24]. That makes it also an interesting candidate for the treatment of other malignancies. However, data concerning gynecological malignancies in general and uterine sarcoma in particular are missing.

Here we attempted to establish a uterine sarcoma cell model for testing vorinostat *in vitro *and *in vivo*. For this purpose MES-SA cell line was used since it has been shown that these cells are tumorigenic in nude mice [1]. In fact, after injecting 5 × 10^6 ^MES-SA cells into nude mice, tumor growth has been induced with 100% efficiency. Our intention was also to use this model as an alternative for endometrial stromal sarcoma. Immunohistochemical comparison of MES-SA and ESS-1 cells proved that these two cell lines are quite similar regarding different cell markers.

In our experiments, both cell lines (MES-SA and ESS-1) expressed different HDACs and responded similarly to the treatment with vorinostat. That might make endometrial stromal sarcomas and uterine sarcomas in general potential candidates for treatment with vorinostat and/or other HDAC inhibitors. Both our *in vitro *and in *vivo data *clearly indicate that vorinostat is able to significantly reduce MES-SA cell-growth already after a short treatment period and at a dose range used therapeutically in the clinic. Moreover, it has been shown by others that in this concentration range vorinostat is well tolerated and causes only minor side effects in patients [25]. In our experiments we did not observe any pathological changes in the main organs in mice, suggesting that vorinostat may have no pronounced toxic effects during treatment over 21-days. These data correlate well with data from long-term studies in humans, in which vorinostat has been used as a therapeutic agent for cutaneous T-cell lymphoma and some other solid tumors. Reduction of tumor volume for more than 50% in comparison to the placebo group shows the potential of vorinostat in the therapy of uterine sarcomas. Our data also suggest that this reduction of tumor size is not so much the effect of diminished tumor cell proliferation but mainly due to specific apoptotic cell death caused by vorinostat. Descriptions of mechanisms involved in the cell death caused by vorinostat treatment of different cell lines are somehow contradictory and seem to depend on the cell model used. However, it seems obvious that apoptosis as well as autophagy play important roles. Thus, further studies should clarify whether one of these mechanisms excludes the other, or whether they are somehow compensating each other during or after vorinostat treatment.

## Conclusions

In summary, we showed that vorinostat efficiently killed tumor cells and impaired the colony forming ability of uterine sarcoma cells *in vitro*. It also influenced the expression of different HDAC enzymes and p21^WAF1^. *In vivo *experiments showed that vorinostat efficiently inhibited tumor growth in nude mice xenografts by activating apoptosis. On the basis of these data and those presented earlier on endometrial stromal sarcoma cells, we conclude that vorinostat might be a promising candidate for therapy of patients with different types of uterine sarcomas.

## Competing interests

The authors declare that they have no competing interests.

## Authors' contributions

AH designed the study, participated in acquisition, analysis and interpretation of data, performed *in vivo *experiments and drafted the manuscript. FM participated in the design of the study and helped to draft the manuscript. MLK carried out cell culture, FACS experiments and helped to perform *in vivo *experiments. BS carried our immunoassays, performed statistical analysis and helped to perform in vivo experiments. EP participated in the design of the study and helped to draft the manuscript. KZ participated in the design of the study. HD participated in the design of the study and helped to draft the manuscript. All authors read and approved the final version of this manuscript.
